# Sustainable Transformation of Cellulose-Containing Textile Waste into Multifunctional Panels with Tailored FR-Lignocellulosic Fibres

**DOI:** 10.3390/polym16233242

**Published:** 2024-11-22

**Authors:** Hamid Lamoudan, Lahbib Abenghal, Dan Belosinschi, François Brouillette, Patricia Dolez, Raymond Panneton, Cécile Fonrouge

**Affiliations:** 1 CTT Group, Saint-Hyacinthe, QC J2S 1H9, Canada; 2 Innovations Institute in Ecomaterials, Ecoproducts and Ecoenergies (I2E3), Université du Québec à Trois-Rivières, Trois-Rivières, QC G8Z 4M3, Canada; lahbib.abenghal@uqtr.ca (L.A.); francois.brouillette@uqtr.ca (F.B.); 3 Department of Human Ecology, University of Alberta, Edmonton, AB T6G 2N1, Canada; pdolez@ualberta.ca; 4 Centre de Recherche en Acoustique-Signal-Humain, Université de Sherbrooke, Sherbrooke, QC J1K 2R1, Canada; raymond.panneton@usherbrooke.ca; 5 Institut de Recherche sur les PME, Université du Québec à Trois-Rivières, Trois-Rivières, QC G8Z 4M3, Canada; cecile.fonrouge@uqtr.ca

**Keywords:** textile waste, management, circular economy, sustainable construction, papermaking process, fire-resistant fibres

## Abstract

The fashion industry significantly impacts the environment, mainly through the substantial generation of waste textiles fostered by fast fashion business models. This study introduces an innovative approach to textile waste management by recycling waste textiles without the use of chemical or mechanical treatments. Herein, we developed a method adhering to the principles of circular economy to transform these textile wastes into high-quality construction panels using a papermaking process. This method not only provides a sustainable solution to reduce landfill dependency but also enhances resource efficiency in the construction industry. The fabricated panels, composed of a blend of 45% textile waste microfibres and 55% fire-retardant fibres, exhibit several advantageous properties. They feature a low apparent density ranging between 170–180 kg/m^3^ and a low thermal conductivity coefficient of 0.047 W/m∗K at 50 kPa. It revealed that phosphorylated fibres not only provide flame-retardant properties, but they also significantly improve the mechanical properties of the panels. For example, load at break increases from 12.4 to 81.1 N, stress at break from 0.44 to 3.59 MPa, and E-modulus from 29.2 to 198.8 MPa after the addition of these 55% fibres. Moreover, these panels successfully met the criteria set by international standards for construction products satisfying the fire test, EN ISO 11925-2. These characteristics make the panels superior options for sustainable construction materials, offering enhanced fire resistance and insulation properties, which are critical to meet modern building standards. They mark a pivotal step towards sustainable construction and waste reduction in the fashion industry.

## 1. Introduction

Textile manufacturing has become one of the most important industry sectors today, playing a vital part in the economies of many countries [1]. According to forecasts, the textile market’s revenues are expected to exceed 1.36 trillion dollars in 2024, and reach 1.78 trillion dollars by 2029 [2,3,4]. This industry produces over 100 billion garments per year, a number that continues to rise year after year, as people are influenced by fashion and most garments are only worn a few times (seven to ten) before being discarded [5,6,7]. As a result, 92 million tonnes of textile waste end up in recycling facilities every year, some of which are still in good condition and can be reused after minor cleaning and repairs [8,9]. However, only 1% of textile waste is recycled after use to make new garments, while the rest ends up in incinerators or landfills [8]. This presents a serious problem for municipal waste managers, and contributes to the high environmental footprint of textiles. Indeed, studies have shown that this industry alone is responsible for 8–10% of total greenhouse gas emissions and 20% of the world’s wastewater [10,11]. Recycling and reuse of textile waste are good ways to solve this problem. This transition can decrease reliance on natural fibres like cotton, which currently makes up approximately 22% of the world’s annual fibre production [12]. Reducing the use of cotton, which is heavily utilized in garment manufacturing, could help mitigate environmental impacts such as high water usage, pesticide dependency, and extensive land requirements. It is estimated that the production of one kilogram of cotton can require up to 20,000 L of water, which means that the manufacture of a single T-shirt can take 2700 L of water [5].

In general terms, textile wastes are divided into two categories: pre-consumer wastes and post-consumer wastes [13]. The first category is generated during the manufacturing process, while the second is produced by consumers after use. The recycling of these textile wastes can be carried out according to three procedures: biological recycling, mechanical recycling and chemical recycling [11,14]. The appropriate procedure is selected based on the waste fibre content: cellulose-based fibres such as cotton, hemp, viscose, and lyocell; synthetic fibres such as polyamide and polyester; and finally, protein fibres such as silk and wool [7].

Biological recycling involves the transformation of textile wastes into simple molecules, namely water, carbon dioxide, methane, ethanol and ammonia, by enzymes or micro-organisms [15]. However, this process is not widely applicable to textile wastes, as in most cases they contain a blend of synthetic and natural fibres, and micro-organisms and enzymes would need several years to achieve complete decomposition. The decomposition period of synthetic polymers is lengthy, and plastic pollution is a serious environmental challenge in this century [16]. Mechanical recycling is considered the most widespread process, and involves the use of mechanical force to reduce textile wastes to fibres, which are then mixed with virgin fibres to produce yarns and then fabrics. Unfortunately, mechanical recycling tends to damage the structure of the fibres and reduce their length, limiting their use for the manufacture of high-value products [17,18]. Finally, chemical recycling is based on fibre depolymerization using strong acids such as phosphoric acid or sulfuric acid or polymer chain dissolution using organic solvents [19,20]. For instance, S. Miguel et al. [20] have used a two-step procedure to depolymerize cotton fibres contained in textile wastes. This process relies on the use of concentrated sulfuric acid in the first step, followed by a second hydrolysis step using a dilute solution from the same acid for total depolymerization of the cotton cellulose to produce glucose. Glucose solutions with concentrations of about 40 g/L were obtained with a yield of 90%. Palme et al. [21] used solutions of 5–15% sodium hydroxide at 70–90 °C to dissolve polyester cotton blended textiles in order to separate the cotton from the PET (polyethylene terephthalate). Ionic liquids or solvents such as N-methylmorpholine-N-oxide (NMMO), which are more environmentally friendly than what is employed in the viscose process, can also be used to dissolve cellulose fibres and produce cellulose solutions for the production of regenerated cellulose fibres [19,22]. However, the chemical process also has certain disadvantages, such as the use of strong acids which can rapidly damage process equipment, and the difficult recovery of acids and solvents [23]. In addition, they may lead to the severe degradation of the fibres.

To fill this gap, this study presents a simple and efficient method for reusing textile wastes to manufacture new value-added products that are in demand in today’s market. This method is based on the use of a blend of textile waste microfibres and chemically modified lignocellulosic fibres to manufacture fire-resistant panels that can be used in the construction industry to improve the building safety.

## 2. Materials and Methods

### 2.1. Materials and Synthesis

#### 2.1.1. Materials

Textile waste microfibres were collected from domestic laundry appliances (washer and dryer combo) by the researchers. They contained both synthetic (mainly polyester) and cellulose-based fibres (cotton, lyocell, and viscose). In this research, flame-retardant lignocellulose fibres were produced by phosphorylating unbeaten bleached softwood kraft pulp fibres (referred to as KF), which were provided by Kruger Wayagamack Inc. (Trois-Rivières, QC, Canada). The phosphorylation process utilized 1-decanol, polyphosphoric acid, phosphorus pentoxide (from Sigma-Aldrich, St. Louis, MI, USA), and urea (from Alfa Aesar, Ward Hill, MA, USA).

#### 2.1.2. Synthesis

**Figure 1:**
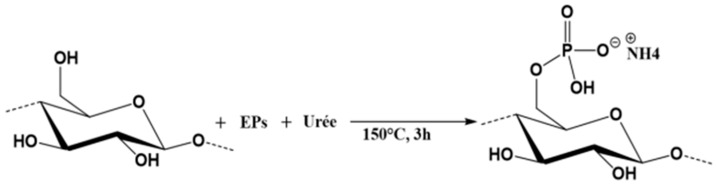
Phosphorylation reaction scheme of lignocellulosic fibres using the phosphate ester/urea system.

The KF were phosphorylated using the previously synthesized phosphate ester (EPs) (Figure 1). This modification was carried out in molten urea, with a molar ratio of urea to phosphate ester and anhydroglucose unit from cellulose polymer set at 17:3:1. The phosphorylation reaction is a commonly used process in our research group and is exhaustively described in several previous papers [24,25]. The phosphorylated fibres (PKF) used in this study had a phosphorus content of 11.2%.

### 2.2. Textile Waste Panel Preparation

**Figure 2:**
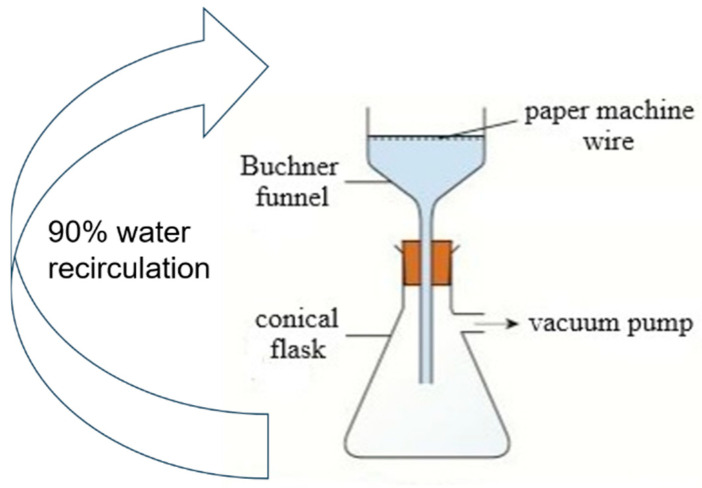
Wet fibre pad formation.

An empiric method was used for the production of prototypes (Figure 2). The panels were prepared by adding 16 g of textile waste microfibres (45%) and 19 g of SF fibres (55%) in 2 L of tap water, followed by dispersion in a standard laboratory disintegrator operating at 3000 revolutions per minute for a duration of 15 min. Subsequently, the fibre suspension was poured into a Buchner funnel containing a paper-making wire mesh with a diameter of 150 mm. A wet fibre pad was formed by applying a 500 mm Hg vacuum to the system. Finally, the wet structure was taken and dried in an oven at 110 °C for 2 h. A series of 10 panels was manufactured. For panels #2 to #10, 90% of the white water was recovered from the previous test and reused in the process. For this study, only the last five panels were kept for further analyses and investigation. The first five panels were excluded to allow the dissolved and colloidal substances to accumulate in the process water by recirculation [26].

### 2.3. Fibre Length Distribution

An L&W Fibre Tester optical device (ABB, Brampton, ON, Canada) was used to determine the mean arithmetic fibre length and width, and the length weighted percentage of fine fibres (less than 0.2 mm in length). A fibre sample of approximately 0.2 g was disintegrated in 200 mL of water with a mixer until the fibres were completely individualized. The device requires a total of 3000 to 5000 fibres to achieve a consistent statistic for each test. Five measurements were conducted for each fibre sample and used to calculate the mean and standard deviation.

### 2.4. Zeta Potential

The Zeta potential of the aqueous textile waste and phosphorylated kraft fibres (PKF) suspensions was determined by simultaneously measuring their conductivity, pressure and streaming potential with a Mutek SZP-06 analyzer (BTG Americas, Norcross, GA, USA). Samples were drawn into the suction tube under vacuum, forming a fibre plug within the measuring cell. Water flow passing through the fibre plug displaced mobile charges from the shear plane, generating a streaming current and thereby establishing a measurable potential difference across two electrodes. The conductivity of the suspensions was adjusted to 0.1–7 mS/cm with 0.1 M KCl to generate a readable streaming potential and ensure reproducibility.

### 2.5. Surface Charge

The surface charge of the textile waste microfibres was evaluated using a Mutek PCD-03 analyzer (BTG Americas, Norcross, GA, USA) equipped with an automatic titrator [27]. Polyelectrolyte titration involves the use of standard polydiallyldimethylammonium chloride (poly-DADMAC, positively charged) and poly(vinyl sulfate) potassium salt (PVSK, negatively charged). Initially, 0.2 g of fibres were added to 50 mL of distilled water. The suspension was then stirred using a magnetic stirrer. Next, an excess of 0.001 N poly-DADMAC was introduced (10 mL for textile waste microfibres and 100 mL for phosphorylated fibres), and the mixture was stirred for 30 min at room temperature. The suspension underwent gravitational filtration using a 200 mL volumetric flask, funnel, and a 202 grade filter paper. Finally, 10 mL of the resulting filtrate were extracted from the volumetric flask and poured into the measuring cell of the PCD-03. The excess of poly-DADMAC in the filtrate was titrated with the negatively charged PVSK standard 0.001 N solution. The surface charge density of the fibre samples was calculated as the difference between the total amount of poly-DADMAC added and the excess amount found after adsorption on the fibre sample.

### 2.6. Nitrogen Content

The Kjeldahl method [28] is frequently employed to quantitatively assess the nitrogen content of organic compounds. Samples are initially digested at elevated temperatures in a strong acid and salts, such as concentrated sulfuric acid and potassium sulfate, with a catalyst like copper sulfate, to convert organic nitrogen into ammonia. Subsequently, the solution is cooled down and neutralized with an alkaline solution, releasing ammonia in gaseous form. The ammonia is then captured in a weak acid solution like boric acid through distillation. The nitrogen amount in the solution is determined by titration with a standard solution of strong acid. This method enables the evaluation of nitrogen content in both textile waste microfibres and SF fibre samples.

### 2.7. Fibre Composition

As the textile waste microfibres contained a blend of synthetic and cellulose based fibres, an empirical method was developed to assess their chemical composition. Cupriethylenediamine (Cuen) was used as a solvent for the cellulosic fibres. Initially, a sample of 0.3 g of fibre was inserted into a 150 mL glass bottle; 100 mL of 1M Cuen were then added to the fibres and the solution was well mixed for 60 min. Finally, the undissolved fibres associated with the synthetic fraction were separated through filtration with a silicone filter paper, thoroughly washed with water, dried, and weighed. The synthetic fraction was calculated by dividing the weight of the undissolved fibres by the total weight of the sample. The result was expressed as the average of three measurements.

### 2.8. Morphological Examination

Scanning electron microscopy (15 kV, variable pressure) coupled with energy dispersive Xray spectroscopy (SEM/EDX, Hitachi High-Tech Corporation, Tokyo, Japan, SU1510 with Oxford X-max 20 mm^2^) was used to track structural changes occurring at the fibre surface during the phosphorylation reaction, and to evaluate the phosphorus content and its distribution on the surface of treated fibres.

### 2.9. FT-IR Spectroscopy

Transmission mode FT-IR spectra were recorded to identify the functional groups of the fibre composing our panel. The analysis was performed using a Thermo Scientific is10 FTIR spectrometer, (Thermo Fisher Scientific, Waltham, MA, USA) over a spectral range of 4000 to 400 cm^−1^, with a resolution of 4 cm^−1^ and 32 scans per sample.

### 2.10. Thermal Conductivity

The thermal conductivity of the textile waste panels was assessed with a MTPS effusivity tester (C-Therm, Fredericton, NB, Canada), following the standard testing method ASTM D7984-21 [29]. The samples were conditioned at 20 °C and 65% humidity for at least 24 h prior to analysis. The tests were conducted in the same environment. Pressure of 50 kPa, the maximum value recommended by the ASTM D7984-21 testing method, was applied to the samples using a pressure foot to ensure good contact between the sample and the temperature sensor. The result was expressed as the average of six replicates.

### 2.11. Open Porosity and Bulk Density

Open porosity is defined as the fraction of volume that is occupied by the fluid in the interconnected porous network. The bulk density is the in-vacuum density of the porous aggregate. Both properties are measured using a bulk volume of 196 cm^3^ with a Porosity/Density meter (Mecanum Inc., Sherbrooke, QC, Canada) according to the procedure outlined in [30]. The average of three measurements on each panel was reported for subsequent interpretation.

### 2.12. Tortuosity

Tortuosity is here defined as the square of the ratio of effective acoustic path length over direct path length through the porous medium. It was assessed using an ultrasonic Transmission Tortuosity Meter (Mecanum Inc.), according to a previously developed procedure [31]. The testing frequency range spanned from 100 kHz to 1000 kHz, with a sampling interval of 50 kHz. The average of three measurements on each panel was reported for subsequent interpretation.

### 2.13. Airflow Resistivity and Permeability

Airflow resistivity (R) was determined using the Airflow Resistance Meter (Mecanum Inc.) according to the ISO 9053-91 standard [32]. A panel sample with a 100 mm diameter was inserted into the instrument channel, and the edges were sealed with petroleum jelly. The average of three measurements on each panel was reported for subsequent interpretation. Note that the corresponding airflow permeability (P) of the material is obtained from the relation P = µ/R, where µ is the dynamic viscosity of air.

### 2.14. Viscous and Thermal Characteristic Lengths

The viscous and thermal characteristic lengths are the average macroscopic dimensions of the cells related to the viscous and thermal losses, respectively, of acoustic waves propagating in the material. The former may be seen as an average radius of the smaller pore channels, and the latter as the average radius of the larger pore channels. Together with open porosity, tortuosity, and airflow resistivity, they are used in acoustical models to predict the sound absorption coefficient and the sound transmission loss of open-cell porous media [33]. The identification of these two lengths was performed using the inverse procedure described in [34]. The average of five samples of 44.44 mm in diameter was reported for subsequent interpretation.

### 2.15. Resistance to Ignition

The flammability of the textile waste panels was assessed using a vertical flammability test setup following EN ISO 11952-2 [35]. A propane gas flame with a height of 20 mm was applied on the specimen surface at a 45-degree angle for 15 s. Following the removal of the flame, the panels were inspected to determine the length of flame spread from the point of flame application. Based on EN 13501-1 [36], if the flame spread is less than 150 mm, the sample is categorized as class E material.

### 2.16. Tensile Strength Properties

Paper handsheets made from 100% phosphorylated fibres, 100% textile waste, and 55/45% mix of phosphorylated fibres and textile waste were prepared according to the TAPPI T 205 method “Forming handsheets for physical tests of pulp”. Two major changes were made to adapt the method to our specific requirements: (1) The basis weight of paper was set at 500 g/m^2^. (2) The drying was carried out at 110 °C in a laboratory dryer for paper.

The samples of paper were cut and tested to tensile strength according to the TAPPI T 220 method “Physical testing of pulp handsheets”. The samples were preconditioned for 24 h at a temperature of 23 °C and a relative humidity of 50% before analysis. 10 tests for each sample were performed and the result is expressed as their average. The tensile index was measured with an Instron 4201 (Instron, Norwood, MA, USA) (TAPPI/ANSI T 494 om-22) [37].

## 3. Results and Discussion

### 3.1. Characterisation of Fibres

The fibre length, width and fines content are shown in Table 1. The PKF are on average at least twice as long as the textile waste microfibres. The PKF is produced from softwood kraft pulp fibres, so a length between 1 and 3 mm was expected [38]. The average diameter of PKF fibre is also significantly larger, almost 30% higher than the microfibres from textile waste. As expected, the fines content of PKF is very low. The fibre is also associated with a narrow size distribution and long and thick fibres. On the other hand, the microfibres from textile waste are characterised by a high fines content and short fibres. From a papermaking perspective, mixing the long and thick fibres of PKF with the short and thin textile waste microfibres creates an opportunity to develop uniform and dense panel structures, with the short and thin textile waste fibres filling the voids between the longer PKF. However, as papermaking is a wet-web formation process, a high amount of fines might increase the risk of fines accumulation in the process water. In this case, the high fines content in the textile waste microfibres did not present a significant problem as most of the fines were physically retained into the panel structure; a closed loop process was made possible, where more than 80% of the process water was reused.

Table 1 also shows the electrostatic properties of the fibres in water. Both types of fibres were electronegatively charged. The PKF fibres exhibited a surface charge density at least 10 times higher than the textile waste microfibres. This can be attributed to the high density of grafted phosphate moieties on the surface of PKF that dissociate and ionize very easily in water. Despite the large difference in charge density between the PKF and the textile waste fibres, the zeta potential of the PKF fibres was only slightly more negative than that of the textile waste microfibres. The charge on the surface of a particle is concentrated in two physical zones [39]: a compact or fixed layer and the diffuse layer. The Zeta potential is the measurement of the potential at the slippage plan that separates these two layers [40]. A high Zeta potential value means the charges are concentrated in the diffuse layer while a low value means they tend to accumulate in the fixed layer. In the case of the PKF fibres, the high density of charges on the surface and relatively low Zeta potential indicate that the charges tend to accumulate in the fixed layer. On the other hand, the fact that the textile waste microfibres are characterised by low values of both surface charge density and Zeta potential points to surface charges mainly concentrating in the diffuse layer. The negative Zeta potential values measured for both types of fibres suggest a certain degree of electrostatic repulsion between the fibres suspended in water, with a greater stability and a lower tendency to aggregate in the case of PKF. In papermaking, dissolved and colloidal substances tend to accumulate as anionic contaminants in the process water. To monitor and semi-quantitatively assess this “anionic trash”, papermakers measure the Zeta potential and the cationic charge demand of the white water. A high cationic charge demand coupled with a strong negative Zeta potential signals a significant buildup of anionic trash, indicating an important contamination of the white water. In addition, the low values of Zeta potential and surface charge density of the textile waste microfibres suggest minimal dissociation and solubilization in water. As the electrostatic properties of fibres are caused by the dissociation or solubilization of some substances/fractions [41], this is a clear indication the microfibres from textile waste are relatively clean and pose a low risk to water contamination despite their heterogenous composition of synthetic and natural fibres. In textile waste microfibres, the nitrogen (N) content is a clear indicator of a heterogeneous composition, suggesting the presence of various fibre types, including synthetic, cellulosic, wool, hair and even certain dyes. This diverse makeup is reflected in the relatively high nitrogen content of about 1.6%, as measured by the Kjeldahl method. In specialty fibres (SF), nitrogen is a fundamental component of their chemical structure, often combined with grafted phosphate groups. This combination of phosphorus (P) and nitrogen in SF creates a synergistic effect that significantly enhances their fire-retardant properties.

### 3.2. Properties of Panels Made with Textile Waste Microfibres

#### 3.2.1. Structural Properties

**Figure 3:**
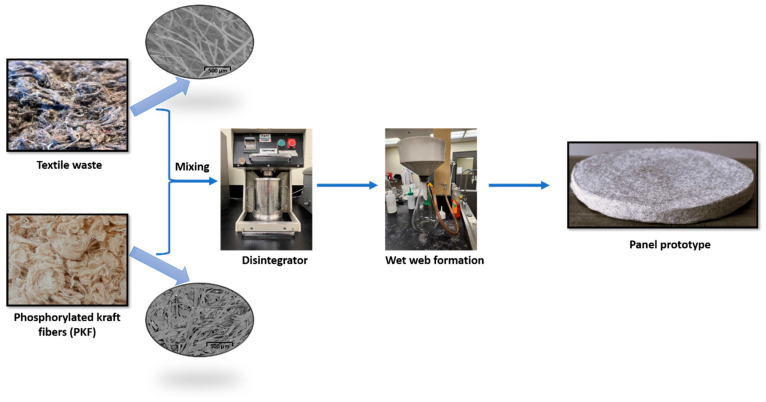
Manufacturing process of panels from textile waste microfibres.

The panels made from the textile waste microfibres and PKF fibres (Figure 3) showed good rigidity when assessed qualitatively and were evenly colored despite their heterogeneous composition. Table 2 shows that the prototype panels reached typical values for building insulation panels of mass per unit area (about 1800 g/m^2^) and thickness (about 10 mm). However, the standard deviation for these parameters was quite high. This can be attributed to the manual nature of the manufacturing method used. The structures were lightweight, with a bulk density of about 180 kg/m^3^, and were highly permeable, with about 86% open voids. The pores were quite small, about 5 μm in diameter. Additionally, the path that the fibres follow within the panels was quite convoluted, with a tortuosity of 2.

The panels made with the blend of textile waste microfibres and PKF fibres exhibited a thermal conductivity of 0.047 W/m∗K, which is almost 50% higher than the values reported in the literature for similar structures made from wood fibres and/or synthetic fibres [42]. This can be attributed to the application of 50 kPa of pressure during the test, which was required to ensure appropriate contact between the MTPS sensor and the surface of the panel. This applied pressure compressed the panel structure and removed the air from the panel voids, thus reducing the overall thermal insulation of the panel. As a result, the value recorded for the thermal conductivity corresponds to the constitutive materials of the panel rather than the structure of the panel itself [43].

#### 3.2.2. Fibre Morphology—SEM/EDX

**Figure 4:**
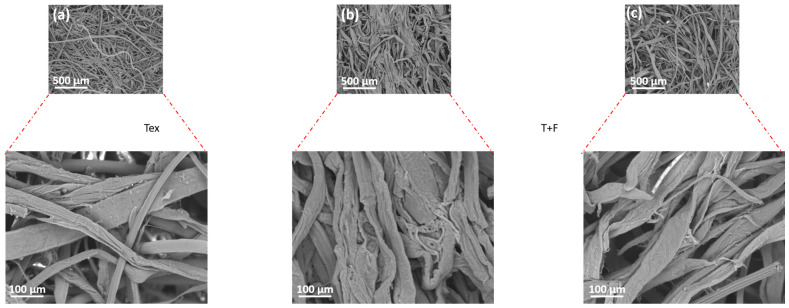
SEM images of (**a**) textile waste microfibres, (**b**) PKF and (**c**) Panel made from a mixture of textile waste microfibres and PKF.

The morphology of the fibres inside the prototype panel was compared to that of the constitutive fibres in their original condition using SEM (Figure 4). In Figure 4a, the textile waste microfibres appear as short and thin flattened strands with a smooth non-fibrillated surface. The PKF fibres were observed to be longer and thicker than the textile waste microfibres from Figure 4b, in agreement with the length and diameter values reported in Table 1. Their surface was also smooth without a significant presence of fibrils. Figure 4c depicts the microstructure of the composite panel. The fibres here are visibly more twisted and heterogenous in size, with a mix of long and short units. Despite the large polydispersity in fibre sizes, the resulting panel shows a homogenous texture with a relatively uniform distribution of fibres and voids throughout the structure. This may be caused by the entanglement of long and short fibres during the panel formation process. Overall, the SEM images reveal slight differences in the morphology and structure of the two types of fibres. These differences allow for a good mixing and interconnection of fibres into a more homogenous panel structure.

The EDX results are presented in Table 3 below.

The PKF sample contains significant amounts of oxygen (44.3%), phosphorus (4.4%), and nitrogen (7.8%). The presence of phosphorus in such quantities is expected, as the PKF sample is obtained through grafting of phosphate moieties on cellulosic fibres. The presence of nitrogen is also beneficial, as it works synergistically with phosphorus to induce flame-resistant properties in the fibres. The high content of phosphorus and nitrogen in PKF confirms that the phosphorylation treatment was effective. The textile waste sample, on the other hand, has a high carbon content and a residual nitrogen content, which is consistent with its composition. Textiles are typically made from carbon-rich organic polymers, such as polyester and cotton, which was further confirmed through Cuen analysis. The low nitrogen content in the textile waste suggests the presence of some contaminants such as wool, hair, or certain nitrogen-based dyes like azo dyes. A low nitrogen content in the textile waste has also been confirmed through Kjeldahl analysis. As expected, the value distribution of elements in the panel prototype made from the mixture of textile waste and PKF lies between those of textile waste and the PKF samples. The carbon content (53.9%) is lower than that of the textile waste but higher than PKF, according to the composition of the mixture (45/55%, *w*/*w*). The presence of nitrogen and phosphorus in larger quantities than in textile waste but smaller than in the PKF sample suggests a potential fireproof behavior induced in the panel prototypes.

#### 3.2.3. FTIR Spectroscopy

**Figure 5:**
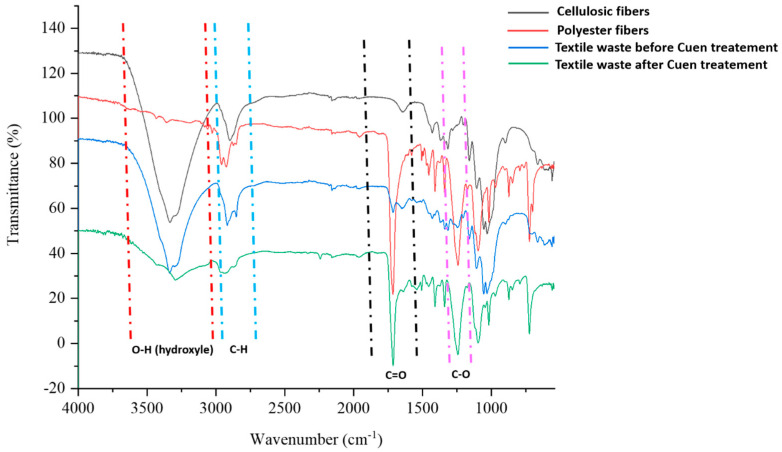
FT-IR spectra of cellulosic fibres, polyester fibres, and textile waste before and after Cuen treatment.

Figure 5 shows the FT-IR spectra of four samples: cellulosic fibres (as standard), polyester fibres (as standard), and textile waste before and after Cuen treatment.

The FT-IR spectra reveal several characteristic peaks. There is a broad, strong peak around 3500–3300 cm^−1^ corresponding to hydroxyl (O–H) groups, which are prevalent in cellulosic fibres and textile waste before Cuen treatment, but it is missing in polyester fibres and vanishes almost completely in the sample of textile waste after Cuen treatment. An important peak is observed in the 1300–1000 cm^−1^ region due to the C–O stretching vibrations. This peak is strong in intensity in case of ester bonds present in polyester fibres and textile waste sample after Cuen treatment but rather weak in intensity in case of cellulose fibres and textile waste sample before Cuen treatment. In synthetic polymers, like polyester fibres and textile waste sample after Cuen treatment, strong peaks appear around 2900 cm^−1^ (C–H stretch) and 1700 cm^−1^ (C = O stretch). These peaks are weak or undefined in case of spectra of cellulose fibres and textile waste before Cuen treatment. In conclusion, Cuen treatment of textile waste causes an almost complete disappearance of O–H stretching peak due to cellulose dissolution and a huge increase of C = O stretching peak due to polyester fibre isolation. The treatment with Cuen solution proves to be effective in determining the composition of the textile waste.

#### 3.2.4. Acoustical Properties

**Figure 6:**
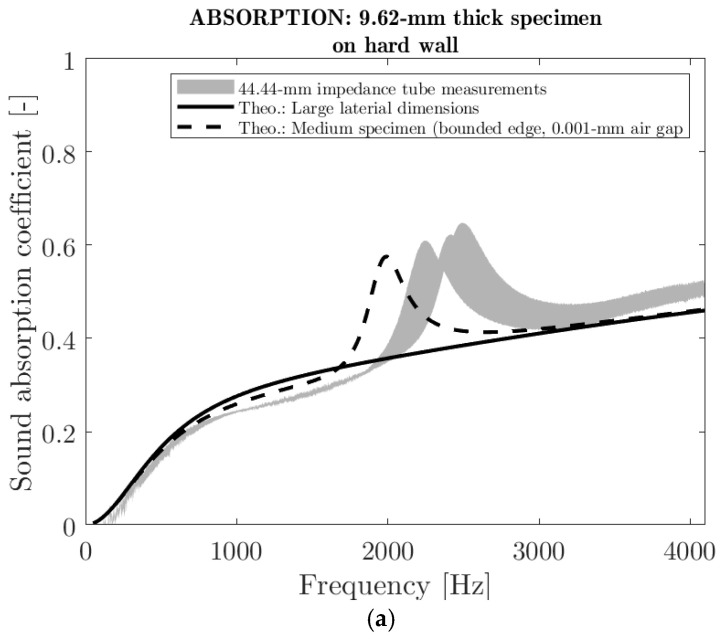

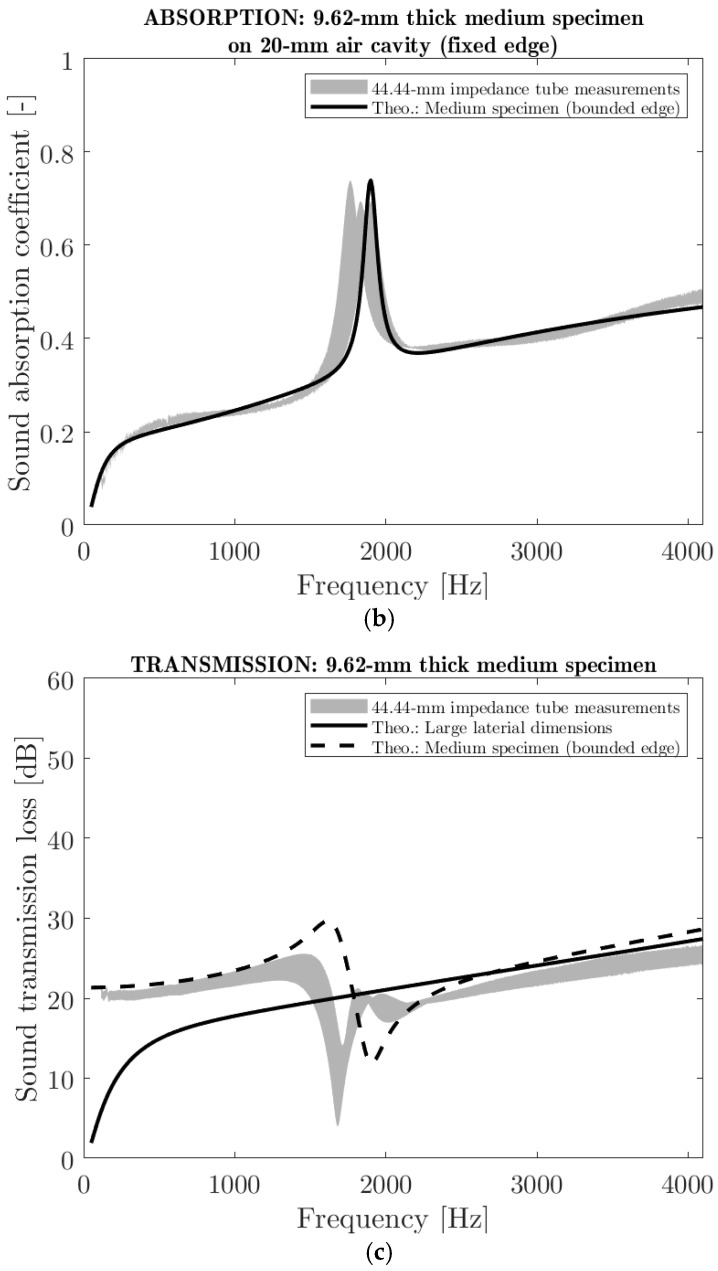
Sound absorption coefficient and transmission loss of panel prototypes (**a**) Normal incidence sound absorption coefficient on hard wall. (**b**) Normal incidence sound absorption coefficient on 20-mm air cavity backed by hard wall. (**c**) Normal incidence sound transmission.

Both the sound absorption coefficient and the sound transmission loss of the manufactured panels were assessed. Figure 6 shows the acoustic performance of a panel of 9.62 mm mean thickness in terms of sound absorption and transmission loss at normal incidence over the 100–4300 Hz frequency range. Figure 6a shows the sound absorption coefficient when the panel was placed directly on a hard wall. The absorption coefficient increased from 0.2 to 0.5 when the frequency increased from 500 to 4300 Hz. The curve shows a peak between 2000 and 3000 Hz which is most likely due to a first plate-like resonance (circular plate clamped on its edge). In fact, when a test specimen of 44.44 mm in diameter is installed in the tube of the same size, the axial motion on its edge is limited due to friction. To proof this, a simulation on a 44.44-mm sample with bonded edge (dashed line in Figure 6a), using a poroelastic model [33], provided a similar profile with a peak located around 2000 Hz. In this case, the elastic properties were inversely identified to best fit the results. On the other hand, this peak is not visible in the case of a simulation with a large-size sample (continuous line in Figure 6a). Overall, the panel prototype has low performance in terms of sound absorption (less than 50% of sound absorption). This low performance is attributed to the low airflow permeability (0.2 × 10^−10^ m^2^—see Table 2) due the high compaction of the material. As a result, the panel produced a shielding phenomenon for the sound. The same type of behavior was obtained when a 20-mm air cavity was present between the panel and the hard wall (Figure 6b). Here, the absorption peak is more pronounced, suggesting an even stronger resonance effect likely caused by the air cavity. Figure 6c illustrates the sound transmission loss in decibels (dB) of the panel prototype. The value of the sound transmission loss is close to 20 dB for the entire frequency range tested. As expected, the resonance effect is also present in the sound transmission loss curve and is attributed to the size of the tested specimen.

In summary, the prototype panel excelled in sound insulation but not in sound absorption due to its low airflow permeability. This low airflow permeability is a consequence of the high compression rate of the material that results in small pore channels, which are restrictive to airflow. A lower compression rate would improve sound absorption at the expense of sound insulation. The impact of the compression rate on the physical and acoustical properties of a fibre assembly is discussed in detail elsewhere [44].

#### 3.2.5. Ignitability

The standard specification EN 13501-1 [36] assigns construction products and floor coverings to a specific class based on their propensity to ignite, assessed according to the EN ISO 11925-2 ignitability test method [35]. This test method was used here to evaluate the fire resistance of the panels made from a blend of textile waste microfibres and PKF using the test conditions for class E products, i.e., with a flame exposure of 15 s. Two parameters were assessed: the extent of flame spread during the test and the ability of the panel to maintain its structural integrity.

**Figure 7:**
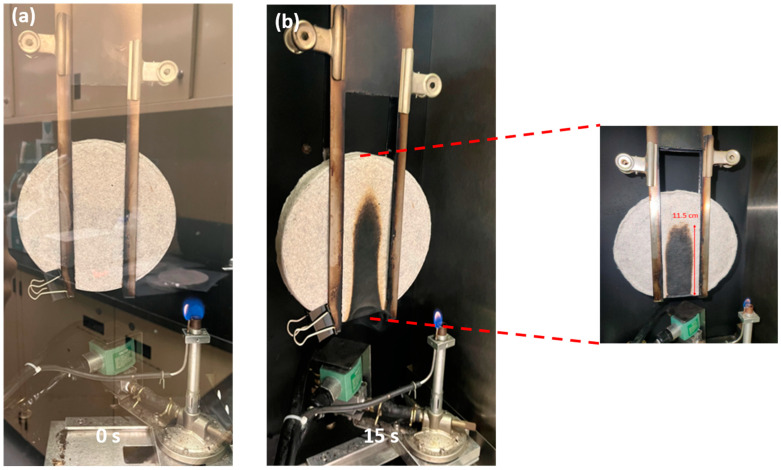
Photographs of prototype panel during the ignitability test (**a**) before and (**b**) after flame exposure.

The tendency of the panels to ignite depends on several factors, but the most important is the chemical composition of the material. In the case of these panels, the textile waste microfibres contain highly combustible materials like cellulose and polyester, while PKF is a fire-resistant phosphorylated fibre. The Cuen analysis revealed that 66% of the textile waste microfibres were cellulose-based; the remaining 33% were most likely polyester. Studies have shown that polyester is less flammable than cotton (cellulose) [45], but cotton/polyester blends can generate more heat and burn faster than fabrics composed solely of cotton [46]. Preliminary trials showed that specimen panels made solely from textile waste microfibres burned and smoked continuously after the 15 s of flame exposure. On the other hand, when 55% PKF fibres were blended with the textile waste microfibres to manufacture the prototype panel, satisfactory results were obtained. When exposed to direct flame, the flame did not spread; it self-extinguished instantly after the withdrawal of the applied flame. Moreover, the panel maintained its structural integrity (Figure 7), the smoke was suppressed in less than a minute and no droplets or incandescent particles fell from the sample. The flame spread was 115 ± 5 mm, significantly less than the 150 mm threshold, indicating that such panels could meet the fire-resistance requirements of class E materials according to EN 13501-1 [36].

#### 3.2.6. Tensile Strength Properties

**Figure 8:**
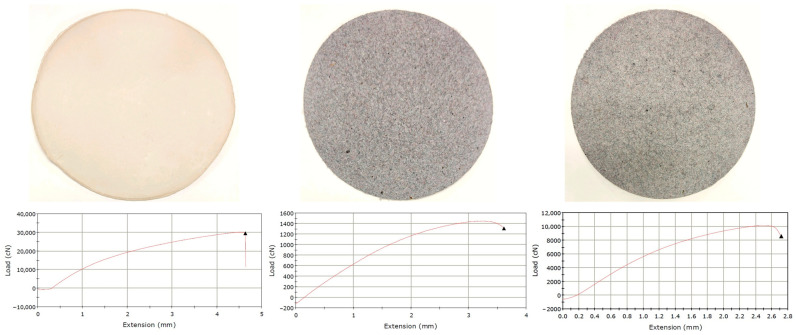
Paper samples and the corresponding stress–strain curves for 100% phosphorylated fibres (**left**), 100% textile waste (**middle**), and 55/45% mix of phosphorylated fibres and textile waste (**right**).

The tensile strength results are shown in Figure 8 and Table 4. As can be easily observed, the tensile strength of paper samples made from 100% phosphorylated fibres is at least one order of magnitude higher than that obtained from 100% textile waste. This can be attributed to the capacity of phosphorylated fibres to create strong bonds in the paper structure due to hydrogen bridges, electrostatic interactions, and even covalent bonds through the crosslinking potential of phosphate moiety. On the other hand, the microfibres from textile waste have an ultralow bonding potential.

The low values recorded for tensile strength suggested a fibre–fibre interaction in the 100% textile waste sample exclusively based on a mechanical interlocking mechanism. Obviously, the paper samples made from a mixture of phosphorylated fibres and textile waste microfibres show tensile strength that places them between the two extremes. In conclusion, the phosphorylated fibres not only confer fire resistance to the panels, but they also bring mechanical resistance to these composite structures.

## 4. Conclusions

The study successfully demonstrates the transformation of microfibres derived from textile waste into multifunctional panels suitable for the construction industry. The proposed method is straightforward and uses technological processes commonly found in the papermaking industry, and provides a very promising alternative to approaches based on complex mechanical or chemical sorting of waste materials. The incorporation of flame-resistant PKF fibres into the panel composition not only imparts fire-resistant properties but also gives stiffness for structural integrity without compromising the lightweight characteristics. These fibres also improve the mechanical properties of the panels, such as load at break, strain at break, stress at break and E-modulus, due to strong hydrogen bonds and electrostatic interactions.

This simple, efficient and low-cost approach makes the method particularly appealing for rapid and large-scale implementation, contributing to environmental protection and more sustainable management of textile resources. Furthermore, the study underscores its significance within a circular-based economy by providing a viable solution for upcycling textile waste into value added products. The study also demonstrates the true power of cooperation. The innovation described here was made possible by bringing together scientists and engineers from various disciplines including materials, textile and papermaking. Finally, this study bolsters the commitment to more ecological and sustainable practices, which are crucial for addressing current and future environmental challenges.

## Figures and Tables

**Table 1:** Characteristics of fibres.

	Fibre Size			Electrostatic Properties in Water		Composition		
	Length (mm)	Width (µm)	Fines (%)	Surface Charge (µeq/g)	Zeta Potential (mV)	Kjeldahl Nitrogen (%)	Synthetic/Cellulosic (%)	Ash at 525 °C (%)
PKF	1.99 ± 0.03	31.9 ± 0.6	0.2	350 ± 15	−55 to −30	5.3	0/100	45 *
Textile waste fibres	0.81 ± 0.02	24.6 ± 0.1	5.6	30 ± 5	−25 to −10	1.6	33/67	0.8

* Char yield due to fire retardant properties of PKF fibres.

**Table 2:** Physical characteristics of panels.

Criteria	Value
Composition, % textile waste microfibres/% PKF	45/55
Mass per unit area, g/m^2^	1864 ± 103
Thickness, mm	9.62 ± 1.02
Density, kg/m^3^	183.3 ± 4.9
Open porosity, %	86 ± 1
Viscous characteristic length, µm	5.4 ± 0.3
Tortuosity	2 ± 1
Thermal conductivity, W/m∗K	0.047 ± 0.003
Thermal characteristic length, µm	60.8 ± 4.8
Airflow resistivity at 0.5 mm/s, N∗s/m^4^	896,260 ± 57,266

**Table 3:** Elemental distribution (atomic weight) as determined by EDX.

Samples	%C	%O	%N	%P
Phosphorylated fibres (PKF)	43.5	44.3	7.8	4.4
Textile waste	65.8	33.4	0.5	N.D *
Mixture of textile waste microfibres and PKF (45/55%)	53.9	40	3.1	2.7

* Not detected.

**Table 4:** Tensile strength properties of papers made from phosphorylated fibres and textile waste.

Sample	Basis Weight, g/m^2^	Thickness, mm	Load at Break, N	Energy at Break, J	Tensile Strain at Break, %	Tensile Stress at Break, MPa	E-Modulus, MPa
Phosphorylated fibres (100%)	500 ± 10	1.15 ± 0.02	292.3 ± 14.6	0.95 ± 0.04	4.67 ± 0.2	26.38 ± 1.31	922.3 ± 46.1
Textile waste (100%)	500 ± 10	2.81 ± 0.11	12.4 ± 0.9	0.03 ± 0.002	3.27 ± 0.21	0.44 ± 0.03	29.2 ± 2.3
Mix of phosphorylated fibres and textile waste (55/45%)	500 ± 10	2.26 ± 0.06	81.1 ± 4.8	0.19 ± 0.01	3.18 ± 0.18	3.59 ± 0.28	198.8 ± 15.9

## Data Availability

The original contributions presented in the study are included in the article, further inquiries can be directed to the corresponding authors.
